# How Can We Increase Postpartum Glucose Screening in Women at High Risk for Gestational Diabetes Mellitus?

**DOI:** 10.1155/2012/519267

**Published:** 2012-03-25

**Authors:** Eeva Korpi-Hyövälti, David E. Laaksonen, Ursula Schwab, Seppo Heinonen, Leo Niskanen

**Affiliations:** ^1^Department of Internal Medicine, Seinäjoki Central Hospital, 60220 Seinäjoki, Finland; ^2^Physiology Department, Institute of Biomedicine, University of Eastern Finland, Kuopio Campus, 70211 Kuopio, Finland; ^3^Institute of Clinical Medicine, Internal Medicine, Kuopio University Hospital, 70211 Kuopio, Finland; ^4^Department of Clinical Nutrition, Institute of Public Health and Clinical Nutrition, University of Eastern Finland, Kuopio Campus, 70211 Kuopio, Finland; ^5^Department of Obstetrics and Gynecology, Kuopio University Hospital, 70211 Kuopio, Finland; ^6^Department of Internal Medicine, Central Finland Hospital District, 40620 Jyväskylä, Finland; ^7^Faculty of Health Sciences, University of Eastern Finland, Kuopio Campus, 70211 Kuopio, Finland

## Abstract

Women with a history of gestational diabetes mellitus (GDM) are at increased risk for diabetes mellitus but postpartum followup is problematic for frequent nonattendance. Our aim was to increase coverage of postpartum oral glucose tolerance tests (ppOGTTs) and examine associated factors. This was a prospective observational study of altogether 266 high-risk women for GDM from 2005 to 2008 in four Finnish municipalities. The groups were as follows: women (*n* = 54) who had previously participated in early pregnancy lifestyle intervention study and high-risk women (*n* = 102) from the same municipalities studied within one-year after delivery. Furthermore, in two neighboring municipalities nurses were reminded to perform a ppOGTT on high-risk women (*n* = 110). The primary outcome was the prevalence of ppOGTT performed and associated factors. Overall the ppOGTT was performed in 35.7% of women. Only 14.7% of women returned for testing to health care centers, 30.9% after a reminder in municipalities, and 82.5% to the central hospital, respectively. The most important explaining factor was a special call or reminder from the central hospital (OR 13.4 (4.6–38.1), *P* < 0.001). Thus, additional reminders improved communication between primary care and secondary care and more attention to postpartum oral glucose testing in primary care are of great importance.

## 1. Introduction

Gestational diabetes mellitus (GDM) implies a substantial risk of later diabetes. Pregnancy seems to identify women who are at risk of developing diabetes later in life. In our study the prevalence of GDM in South Ostrobothnia was 13.0% [1]. About 10% of Finnish women with GDM will develop diabetes over the next 6 years; nearly half of them develop type 1 diabetes and the other half type 2 diabetes [2]. In the meta-analysis of 675,455 women with 10,859 cases of type 2 diabetes Bellamy found that women who have had gestational diabetes have at least a sevenfold increased risk of developing type 2 diabetes mellitus in the future compared with those who have had a normoglycaemic pregnancy. The strength of the association between gestational diabetes and type 2 diabetes and the knowledge that many of the risk factors (family history of diabetes, raised body-mass index, increased age, and Asian and black origin) are the same, suggest that the two disorders might have an overlapping cause [3].

Recommendations from the International Workshop Conference of GDM suggest screening at 6 weeks postpartum using the 75 gram, 2 hour oral glucose tolerance test (OGTT), which should then be repeated at one-year postpartum and then at least every 3 years thereafter [4]. The postpartum period allows for the identification of women at high risk for diabetes and provides an important opportunity for intervention. Type 2 diabetes has been prevented or delayed by lifestyle intervention not only in randomized controlled studies [5], but also in the primary health care setting [6].

Despite the elevated risk for diabetes and recommendations for close followup, the opportunities for postpartum screening and intervention are missed. The variation of screening rate is from 14% in usual care to 60% in a randomized control trial [7]. In a retrospective study a reminder system of automated orders to physicians and telephone and e-mail reminder messages to patients improves the coverage of tested women 50% [8].

GDM strongly predisposes to type 2 diabetes. GDM and type 2 diabetes also share many of the same risk factors, suggesting that the two disorders have an overlapping pathogenesis [3]. Excessive gestational weight gain is a risk, factor for short-term postpartum weight retention and thus overweight in women. In a meta-analysis of >65,000 women Nehring showed that women with a gestational weight gain above recommendations retained 3 kg more weight 3 years postpartum than did those who gained weight within the recommendations [9]. Therefore, women at a high risk for GDM are a high-risk group for diabetes, especially if they have postpartum weight retention.

Our aim in this study was to explore the optimal strategies to increase the amount of postpartum oral glucose tolerance test (ppOGTT). The primary outcome was the prevalence of women at high risk for GDM who underwent an OGTT in the postpartum period in four rural municipalities. In two municipalities we had our previous early pregnancy intervention study group women for preventing GDM [1] and other high-risk women for GDM with usual care, and in two neighboring municipalities high-risk women who received a special telephone call for postpartum glucose testing.

## 2. Methods

This study was a prospective observational multicenter study of postpartum glucose screening in 266 women at high risk for GDM in South Ostrobothnia, Finland. The risk of GDM was estimated during index pregnancy from Apr 2005 to May 2006. We analyzed data from Apr 2005 to Jan 2008 from Seinäjoki Central Hospital and four rural municipalities Kauhajoki, Lapua, Jalasjärvi, and Kurikka.

A database contained clinical, glycemic, and delivery data of all women from four municipalities in the study period. The inclusion criteria for this study were at least one of the follows: (1) BMI >25 kg/m^2^, (2) birth of child >4.5 kg, (3) age over 40 years, (4) family history of diabetes and (5) glucosuria. The risk factors for GDM were reasked in connection with the ppOGTT at the central hospital and in the neighboring municipalities. In the usual care municipalities the information of risk factors was extracted from the self reported questionnaire in the beginning of index pregnancy.

We included also our previous early pregnancy lifestyle intervention study group to the current examination. The protocol was approved by the ethics committee of South Ostrobothnia Hospital District in Seinäjoki, Finland. It was in accordance with Helsinki Declaration. All women participating in ppOGTT gave written informed consent.

### 2.1. Diabetes Care during Pregnancy

According to guidelines in Finland at the time of study, GDM testing was focused on women with risk factors. Diagnosis of glucose intolerance was established by 2-h 75-g OGTT performed between 24 and 28 weeks of gestation. In women with a high risk for diabetes, testing was done in early pregnancy. OGTT testing was also offered if women had polyhydramnion, macrosomia or glucosuria later during pregnancy [10]. The GDM criteria were modified from the World Health Organization as a fasting plasma glucose ≥5.6 mmol/L or 2 h plasma glucose ≥7.8 mmol/L [1]. From 1998 onward, the WHO classified any glucose levels above normal as indicative of gestational diabetes [11].

The health care nurses gave women counseling about healthy lifestyle in the beginning of pregnancy. The dietary and exercise advice were provided both verbally and writing. Women were advised to stop alcohol intake and smoking. The nurse in the health care centers had on average 13 appointments with the women during pregnancy.

In the study group a clinical nutritionist gave dietary advice tailored to each subject individually six times and a physiotherapist gave exercise advice six times. In the control group the women were given general information on diet and physical activity in a single session to decrease the risk of GDM during pregnancy group [1].

After a diagnosis of GDM women were given instructions and advised to perform blood glucose monitoring. Insulin therapy was initiated when fasting capillary plasma glucose exceeded 5.8 mmol/L and postprandial capillary plasma glucose was >8.5 mmol/L in the study period.

All the women were followed in maternal health care in municipalities and all the women gave birth at the Central Hospital of Seinäjoki. During pregnancy the women were seen at the central hospital only if fasting capillary plasma glucose exceeded 5.8 mmol/L or postprandial capillary plasma glucose exceeded 8.5 mmol/L or in the case of macrosomia, polyhydramnion, lack of compliance, BMI >30 kg/m^2^, or some complicating illness.

### 2.2. Postpartum Testing

Women with GDM requiring insulin were asked to continue glucose testing at home after discharge. The women with a diagnosis of GDM were offered an OGTT after one year after delivery based on local instructions.

The high-risk women who underwent a lifestyle intervention during pregnancy were offered an OGTT at the central hospital irrespective of whether they developed GDM. We generated a list of women at high risk for GDM in two neighboring municipalities, took it to the health care nurses and advised them to call the women by telephone for glucose testing in the primary care.

A 75 g 2 h oral glucose tolerance test after overnight fasting for 12 hour was performed one year post partum. Diabetes was diagnosed by either fasting venous plasma glucose ≥7.0 mmol/L or 2 h value ≥11.1 mmol/L, impaired fasting glucose (IFG) tolerance by fasting glucose ≥6.1 mmol/L and impaired glucose tolerance (IGT) by 2-h glucose ≥7.8 mmol/L and <11.1 mmol/L.

### 2.3. Data Collection and Statistical Analysis

Antenatal maternal clinical, glycemic, delivery, neonatal, and ppOGTT data were derived from the database of the central hospital and the health care centers. Variables considered as potentially predictive for a participation in the ppOGTT were analyzed with the statistical package SPSS 19.0 (SPSS, Chicago, IL, USA). Data are presented as numbers and proportion for categorical variables or as means ± SD for continuous variables, respectively. A *χ* ^2^ test or two-sided Mann-Whitney *U*-test was used to test for differences between women who participated in the ppOGTT and those who did not. Differences with *P* < 0.05 were regarded as statistically significant. A multivariable logistic regression model was used to evaluate the association between participation in the ppOGTT and demographic factors, anthropometric and clinical risk factors and special call for ppOGTT. Results for each risk factor are presented as odds ratios (ORs) with 95% CI.

## 3. Results

The baseline characteristics of the 266 women at high risk for GDM are seen in Table 1. The women in the lifestyle intervention study group (*n* = 54) were thinner (*P* = 0.040), they were more often nulliparous (*P* = 0.001) and more frequently had a family history of diabetes (*P* = 0.005) than the other women at a high risk for GDM (*n* = 212). During pregnancy GDM was diagnosed in fewer women (*P* = 0.005) in the lifestyle intervention group (*P* = 0.010) than in the other high-risk women (data not shown). Otherwise there were no differences on tested baseline variables between the lifestyle intervention group and other risk women for GDM in four municipalities.

**Figure 1:**
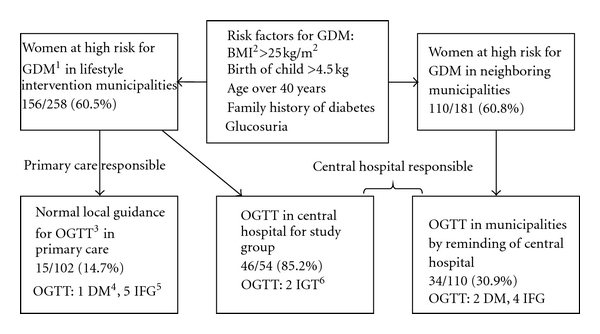
The flowchart of postprandial glucose screening in women at high risk for gestational diabetes mellitus.^ 1^GDM: gestational diabetes mellitus, ^2^BMI: body mass index, ^3^OGTT: oral glucose tolerance test, ^4^DM: diabetes mellitus, ^5^IFG: impaired fasting glucose, ^6^IGT: impaired glucose tolerance.

Women who underwent ppOGTT were more likely to have received a telephone call from the central hospital to remind about the OGTT than women who did not undergo testing (83.2% (79/95) and 49.1% (84/171), respectively, *P* < 0.001). The women in the lifestyle intervention study group returned for testing significantly more often than the other women at high risk for GDM, adjusted OR 2.5 (5.4–29.5), *P* < 0.001. The overall return rate for postpartum testing in women was 35.7% (95/266). In the usual care in the two municipalities in which no reminder call was made the return rate was lowest 14.5% (15/102), 30.9% (34/110) for those receiving the reminder call and in the lifestyle intervention study group 85.2% (46/54) (Figure 1). All of the women who participated in postpartum testing also had OGTT during pregnancy. In the lifestyle intervention study group, 6 women were pregnant again, one had moved away, and one was not willing to undergo testing. In the reminder group 7 women were pregnant, and 68 women refused testing for professional or child care reasons or most because of unknown problems. In the usual care group the reasons could not be assessed.

We tested age, educational status, parity, BMI, weight gain during pregnancy, GDM diagnosed during pregnancy, blood sample of newborn for glucose and risk factors for GDM (BMI >25 kg/m^2^, previous birth of child >4.5 kg, age >40 years, previous history of GDM and family history of diabetes), and special call for ppOGTT. In univariable analysis unadjusted nulliparous women, women with normal weight and higher education returned more often for glucose testing (Table 1).

 The most important explanatory factor was the reminder telephone call for testing either at the central hospital or reminding from the central hospital to the care providers in the health care centers (adjusted OR 13.4 (4.6–38.1), *P* < 0.001). Another statistically significant risk factor was family history of diabetes, (adjusted OR 5.1 (2.1–12.2), *P* < 0.001) (Table 2).

Fourteen women (8.2%) had abnormal glucose tolerance: 3 had DM-, 4 had IGT- and 7 had IFG. Two women out of 4 with IGT diagnosis and one woman out of 7 with IFG-diagnosis had normal glucose tolerance during pregnancy. One woman was obese, BMI 40 kg/m^2^ and she had 2 kg of weight retention postpartum, the other had 8.5 kg of weight retention and the third 6 kg.

## 4. Discussion

GDM is a significant risk factor for the development of diabetes. Early identification of women at high risk for diabetes is critical to prevent or delay onset of diabetes. However, in the real world the opportunities for postpartum screening and intervention are frequently missed. In the present study the inclusion criteria were BMI >25 kg/m^2^, birth of child >4.5 kg, age over 40 years, family history of diabetes and glucosuria. In the usual care in the health care centers, the testing rate was only 14.5%. The return rate was doubled (30.9%) in municipalities in which health professionals from the central hospital reminded the nurses in health care centers to call the high-risk women for glucose testing. The return rate for postpartum testing of women at high risk for GDM was highest (85.2%) in those who participated in the lifestyle intervention study group. Thus a simple reminder from the central hospital was the most important factor explaining better compliance with ppOGTT. To our knowledge there are no earlier reports of postpartum testing women with risk factors for GDM without GDM diagnosis.

The overall return rate for postpartum testing was 35.7% (14.5%, the usual care; 30.9%, after the reminder call; 82.5%, the study group) in the current study. The frequency of follow-up with an OGTT was 33.7% in a retrospective cohort study of women with previous GDM (*n* = 745) in New York [12]. The overall return rate was 51.1% (79.6%, Kiel; 65.0%, Bonn; 23.4%, Berlin) in a German prospective multicenter study of 605 women with GDM [13]. In the Canadian study the postpartum reminding system doubled screening rate from 14% to 28% in the usual care [7]. This result is in accordance with our study. All the Canadian women had GDM, whereas in our study 21.4% (57/266) had GDM, and the rest were at high risk for GDM. However, 42% (11/26) of those women who had GDM returned for glucose testing in usual care. All (4/4) of the women with GDM who had undergone in the lifestyle intervention and 33% (9/27) of the women with GDM who were given a reminder telephone call took a postpartum OGTT. It is worth noting that 2 women with IGT and one woman with IFG had normal glucose tolerance during pregnancy. One woman was morbidly obese and the rest had excessive weight retention one year after pregnancy. Gestational diabetes and type 2 diabetes have the same risk factors, and overweight and weight retention after index pregnancy also seems to be important predictive factors for impaired postpartum glucose tolerance.

Hunt has identified in a prospective cohort study of 707 women several factors associated with postpartum screening: fewer children, lower fasting blood glucose levels at GDM diagnosis, and no insulin treatment in pregnancy [14]. Thus the women who returned for postpartum glucose screening had less severe GDM than women who failed to return. The same tendency was observed also in the current study: over half (58% 33/57) of women with GDM and (57% 12/21) of women with insulin therapy did not attend for testing. In an observational German study Schaefer-Graf examined the association between an abnormal postpartum OGTT and four risk factors: body mass index ≥30 kg/m^2^, gestational age at diagnosis <24 weeks, 1 h antenatal glucose value >11.1 mmol/L and insulin therapy. Women with two or more risk factors had a high risk for an abnormal ppOGTT, and 86% of postpartum diabetes was diagnosed within this group [13].

Even though clinicians are aware that women with GDM are at high risk of developing type 2 diabetes they do not routinely screen patients. Providers identified poor communication between primary care providers and obstetric and gynecology care providers as a major barrier to screening [15]. The current study and the other reminding system studies [7, 16, 17] shows that we have other barriers as well. Feelings of emotional stress due to adjusting to a baby and the fear of receiving a diagnosis of diabetes at the visit were identified as key barriers in a small interview study of 22 women [16]. Child care availability and desire for checkup were among the key facilitators to screening. The fear of receiving a diagnosis of diabetes may have explained in the current study why overweight, multiparous and less educated women frequently did not attend. This may also be a reflection of a “healthy cohort” effect, in which individuals who are more health-conscious are more likely to seek treatment or followup. On the other hand guidance during pregnancy in the intervention group seems to increase the likelihood for postpartum testing.

One of the strengths of this study is that it was performed in a community-based setting in a rural area. This is the first study where the women with risk factors for GDM were called for postpartum glucose testing. The limitation of this study is the small number of women. The Electrical Medical Record (EMR) covers now the study area and improves the possibilities for expanding the system of reminding by telephone. Timing the testing six months after delivery when breastfeeding is finished and child care organized may remove barriers.

## 5. Conclusion

We should improve the communication between primary care providers and obstetrics and gynecology care providers and endocrinologists and develop a reminding system for primary care. A lifestyle intervention during pregnancy and the knowledge that diabetes can be prevented may encourage the women to participate in postpartum testing.

## Figures and Tables

**Table 1:** Characteristics of the women at high risk for gestational diabetes mellitus during the index pregnancy.

Characteristics	No postpartum glucose screening (*n* = 171)	Postpartum glucose screening (*n* = 95)	*P* ^1^
Age (years), mean ± SD	30.1 ± 5.7	30.2 ± 5.7	0.968
<25 (%)	19.3 (33/171)	18.9 (18/95)	
25–35 (%)	63.2 (108/171)	61.1 (58/95)	
≥36 (%)	17.5 (30/171)	20.0 (19/95)	
Educational status			0.044
Higher education (%)	8.8 (15/171)	26.3 (25/95)	
Other education (%)	91.2 (156/171)	73.7 (70/95)	
Parity			0.001
Nulliparous (%)	25.1 (43/171)	45.3 (44/95)	
Multiparous (%)	74.9 (128/171)	53.7 (51/95)	
Body mass index (kg/m^2^), mean ± SD	28.2 ± 5.4	26.7 ± 4.7	0.024
≤25.0 (%)	26.3 (45/171)	38.9 (37/95)	
25.1–30.0 (%)	45.0 (77/171)	45.3 (43/95)	
30.1–35.0 (%)	19.9 (35/171)	9.5 (9/95)	
>35.0 (%)	8.2 (14/171)	6.3 (6/95)	
Weight gain (kg), mean ± SD	11.5 ± 6.5	12.0 ± 5.8	0.453
≤11.5 (%)	50.9 (87/171)	46.3 (44/95)	
11.6–16.0 (%)	26.9 (47/171)	28.4 (27/95)	
>16.0 (%)	21.1 (37/171)	24.2 (23/95)	
GDM diagnosed (%)	19.9 (34/171)	24.2 (23/95)	0.385
Insulin therapy during pregnancy (%)	6.4 (11/171)	10.5 (10/95)	0.235
Blood sample of newborn for glucose (%)	36.3 (62/171)	48.4 (46/95)	0.049
Risk factors for GDM			
BMI >25 (kg/m^2^) (%)	78.9 (135/171)	67.4 (64/95)	0.037
Previous birth of child >4.5 kg (%)	5.3 (9/171)	1.1 (1/95)	0.102
Age >40 years (%)	3.5 (6/171)	3.2 (3/95)	1.000
Previous history of GDM (%)	18.1 (31/171)	14.7 (14/95)	0.480
Family history of diabetes (%)	18.1 (31/171)	52.6 (50/95)	<0.001
Special call for OGTT (%)	49.1 (84/171)	83.2 (79/95)	<0.001

^1^ *P* values (two sided): *χ* ^2^ test or Mann-Whitney *U*-test.

**Table 2:** Multivariable logistic regression model predicting postpartum glucose screening among women at high risk for gestational diabetes mellitus.

	Odds ratio (95% CI)^1^	*P*
Age (years)		
<25	1.00	
25–35	0.73 (0.25–2.15)	0.571
>35	0.75 (0.19–2.98)	0.684
Educational status		
Other education	1.00	
Higher education	0.65 (0.25–1.73)	0.393
Parity		
Nulliparous	1.00	
Multiparous	0.51 (0.20–1.27)	0.149
Body mass index (kg/m^2^)		
≤25.0	1.00	
25.1–30.0	1.07 (0.18–6.36)	0.941
30.1–35.0	1.79 (0.25–12.86)	0.564
>35.0	1.67 (0.19–14.83)	0.646
Weight gain during pregnancy		
≤11.5	1.00	
11.6–16.0	0.71 (0.27–1.89)	0.491
>16.1	0.67 (0.23–1.92)	0.454
GDM diagnosed during index pregnancy	2.23 (0.54–9.26)	0.269
Insulin therapy during index pregnancy	1.31 (0.19–9.12)	0.782
Blood sample of newborn for glucose	1.30 (0.57–2.97)	0.536
Risk factors for GDM		
BMI >25 (kg/m^2^)	0.99 (0.29–3.36)	0.989
Previous birth of child >4.5 kg	0.21 (0.01–3.09)	0.256
Age >40 years	0.95 (0.13–7.06)	0.961
Previous history of GDM	1.63 (0.48–5.52)	0.435
Family history of diabetes	5.09 (2.13–12.12)	<0.001
Special call for OGTT	13.4 (4.64–38.1)	<0.001

^1^Adjusted odds ratios (ORs), and their 95% confidence intervals (CIs) and *P* values.

## Abbreviations

BMI: Body mass index
DM: Diabetes mellitus
EMR: Electrical Medical Record
GDM: Gestational diabetes mellitus
IFG: Impaired fasting glucose
IGT: Impaired glucose tolerance
OGTT: Oral glucose tolerance test
ppOGTT: Postpartum oral glucose tolerance test
OR: Odds ratios.
